# Extracts from Foreign Journals

**Published:** 1896-01

**Authors:** 


### EXTRACTS FROM FOREIGN JOURNALS.

---

THERAPEUTICS OF SERUM AGAINST TETANUS. It has been established that the serum of animals which have been inoculated against tetanus and diphtheria has at the same time a preventive and a curative effect, and can be used as well for preventive inoculations as for treatment of the disease. In the case of tetanus the desired immunity takes place immediately after the injection of the serum, the period of immunity lasting from two to four weeks. The cure is the surer the earlier the injection takes place after the artificial transferrence of the specific microbes, respectively, of the poisons secreted by them. If, however, the injection is made after the natural rigidity (or spasm accompanied by rigidity) has set in, there arises a very serious condition. Although it is possible, in the case of diphtheria, to take the disease in hand at the beginning of its development which precedes poisoning, this is not possible in tetanus, because visible symptoms of the disease do not appear until several days after actual poisoning has set in; and in this state, *i. e.*, when tetanus is no longer to be mistaken, an injection of serum proves to be wholly without effect. Since, then, serum affords no relief after tetanus has broken out in its natural way, it is only possible to use it as a means of prevention. Veterinarians who practise in neighborhoods where tetanus is frequent might often do well, after accidental injuries or operations, to make two injections of serum within fourteen days, merely as a preventive. They could then feel assured that the animal which had been injured or operated upon would not certainly within six to eight weeks fall a prey to tetanus. The injection can be made at the arch of the neck or behind the shoulder-blade. The serum which Nocard dispenses retains its effectiveness for at least six months, provided the phial is not exposed to light or heat and is not opened until the injection is to be made.—*Excerpt from an article by Nocard in the Receuil de Méd., reprinted in the Theirärstliches Centralblatt,* November 15, 1895.

THE SPREAD OF DIPHTHERIA BY HOUSE CATS. The *British Medical Journal* contains the following highly interesting statement: "At the last diphtheria epidemic in Brighton, all the cases occurred in Elm, Grove, and Southover Streets. Everything pointed to the fact that some local cause must be present here. Grown people as well as children were afflicted with the disease. The milk could not be the cause, because the patients had obtained that commodity from several different sources; in like manner the drainage in the houses under consideration was of the best, but in every house the house cat had been sick. The disease of the cats consisted in very severe coughing, excretion at the nose, difficulty in swallowing, and emaciation. Further investigation showed that more than one cat in the streets afflicted with diphtheria had suffered from the same disease. The authorities of Brighton felt sure that the cats had transmitted the disease to human beings, and warned the public against them, with the result that further spreading of the disease was prevented."—*Thierärstliches Centralblatt*, November 15, 1895.

Result of Pasteur Inoculations. In regard to the value of Pasteur inoculations, the following statistics, which are taken from a report of the Pasteur laboratory in Stuttgart, give definite information. The Institute dispensed in the year 1894, the first of its existence in Germany, vaccine matter against erysipelas (swine-plague) for 7847 sucking pigs; against anthrax (morbus carbuncularis, charbon, malignant pustule, splenic fever), for 2200 sheep, 2215 cattle, and 4 horses—in all for 12,266 animals. According to the investigations made, the mean death-rate in the different classes before inoculation was, for sucking pigs, 30 to 40 per cent.; for sheep, 20 to 25 per cent.; and for cattle, 10 to 20 per cent. These figures sank, as a result of inoculation, to 0.57 per cent. for sucking pigs, 0.38 per cent. for sheep, and 0.05 per cent. for cattle.—*Wiener Zeitung* (*Vienna News*).

The Sixth International Veterinary Congress in Berne. From the very first, the most favorable prognosis had to be made for the Sixth International Veterinary Congress in Berne, since not alone the most important veterinary questions were proposed for discussion, but the very choice of the place itself was not of the least importance for attendance upon the assembly. Accordingly the number of announcements (from those who proposed to attend) was nearly 700, about half of which, to be sure, came from Switzerland. Germany stood next with 77; Roumania with 64, and France with 55, followed in order. The numbers for the remaining states were: Belgium, 24; Austro-Hungary, 19; Russia, 12; Holland, 12; Italy, 11; England, 4; Denmark, Sweden, Norway, Luxemburg, Greece, Spain, Portugal, America, India, Japan, and Egypt, 1 to 2. The actual number of participants amounted approximately to 300, of whom 256 entered their names in the list of those present.

Corresponding to this important numerical factor, the assembly was in quality very brilliant; in a word, it united a larger number of important men than has ever been seen at a veterinary congress. We must content ourselves here with a mere statement of this fact, because an enumeration of the individual names would far exceed the limits of this article. It is incumbent upon us, however, to remark that the Swiss Federation and canton government were represented by several gentlemen and by the college of professors in the Berne school in full number. The thanks of all present are due to all these gentlemen for the zeal with which they made all the preparations, for the trouble which it cost them, and for the friendly spirit with which they performed the various services.

The Congress had a decided international character, although the German and French were in a preponderating majority. For this reason the proceedings were carried on in German and French. Only a few of the speakers made use of both languages, and the most of the speeches were therefore translated by two interpreters into the other language. This was done with praiseworthy zeal and great dexterity, the only criticism being that, in consideration of the fulness of the programme and the lack of time, a brevity was often imposed upon the interpreters which was not always conducive to a thorough understanding.

The assembly, up to the time of organization, was presided over by the representative of the Swiss federal government—bundesrath—(federal counsellor), Med. Dr. Deutscher. The first speaker was Colonel Potterat, chief of the Swiss veterinary military officer corps. After him Dr. Deutscher, in a long speech which was frequently interrupted by applause, welcomed the assembly and declared in the name of the government that the latter was following all the proceedings with great interest.

Thereupon followed the election of a president. According to the suggestion of Potterat, Chairman of the Committee on Organization, a different president was chosen for each day, and the following men received that honor: Chauveau (France), Lydtin (Germany), von Raupach (Russia), Hutyra (Hungary), Berdez (Switzerland), Müller (Germany); as vice-presidents (two for each day) were chosen: Degive (Belgium), Hirzel (Switzerland), Nocard (France), Perroncito (Italy), Bang (Denmark), Wirtz (Holland), Siegen (Luxemburg), Vasilescu (Roumania), Siedamgrotzky (Germany), Arloing (France), Sperk (Austria), and Cope (England).

The Congress elected as honorary members: Robert Koch (Berlin), Pasteur (Paris), and Röll (Gratz), and these gentlemen telegraphed their thanks for the honor. Noyer (Berne) was elected General Secretary, whereupon Chauveau, amid lively applause, assumed direction of the proceedings, which were opened with a report by Professor Hutyra on the best means of securing international veterinary police regulations. Berdez, Degive, and Perroncito reported on the same subject, and in the debate which followed Raupach, Degive, Leblanc, Lydtin, Berdez, and Nogneura took part. The views in regard to the possibility and result of such regulations differed widely, and the opinion was several times expressed that the discussion of the question could be of little practical worth on account of the fact that there was positively no hope that such measures could be carried through. In spite of this, however, the following resolutions were passed, although it must be said that a part of the assembly refrained from voting:

"*a.* The Congress recommends the establishment of an international service for collecting statistics in regard to diseases and the publication of an international bulletin for diseases.

"*b.* The Congress resolves to present a petition to the Swiss federative government that it take the initiative in calling together an international conference with a view toward securing a convention to consider the cattle-traffic."

On the second day, with Lydtin as presiding officer, the inoculation of mallein was discussed, and Nocard, Schindelka, Foth, and Heyne presented papers. The debate was very lively, and, in consideration of the importance of the subject, extraordinarily interesting. Professor Schindelka vividly described his well-known opinion, which he had arrived at through a long list of experiments. Foth did not differ essentially from him, but made reference to the opposite relation between the temperature of the body at the time of injection and the highest temperature after it. He referred, further, to the necessity of repeated injections and to the need of more extended experiments at the expense of the State. The present unsuccessful results with the mallein reaction are, in his opinion, worthy of consideration. Nocard declared with the approbation of his countrymen that the mallein question had been settled, and that, according to his experiments, mallein is beyond all doubt a sure diagnostic agent. Müller (Prussia) dissented absolutely from this view on the grounds of statistical data. Grünwald (Moscow), Locusteano, Pilarios (Athens), Leblanc, McFadyean (London), Nogneura (Lisbon), Trasbot (Alfort), Hinze (Hague), Lanzilotti (Milan), Stubbe (Brussels), and Degive spoke further on the same subject, whereupon the following resolution was passed with a small majority, thirty-nine voting against:

"*a.* Mallein is a powerful means of securing a diagnosis of equine glanders in cases suspected of this disease.

"*b.* The systematic use of mallein in herds which are affected with glanders is the surest means of rooting out the disease."

In spite of this, however, a motion of Foch's, which found great favor in the eyes of the less enthusiastic advocates of mallein (Chauveau, Leblanc, Arlonig, Müller), was carried without opposition:

"The different governments are requested to place means at the disposition of veterinarians in order, by means of experiments which will be free from criticism, to settle the question finally in regard to the worth of mallein injections as a regulation of the veterinary police, it being recommended that a number of horses be artificially infected with 'malleus humidus' and treated with mallein."

The debate about tuberculin as a diagnostic, in which Bang (Denmark) gave the first report, was no less interesting. Bang is a warm advocate of tuberculin-inoculation, and holds that the battle against tuberculosis will last for many years, but, nevertheless, in the end lead to victory. Hess and Guillebeau are less enthusiastic in regard to the tuberculin reaction and hold the not uncommon view that miliary tuberculosis can arise through injection of tuberculin. After a rather warm debate, in which many speakers took part, a motion of Bang and Nocard, which runs as follows, was carried:

"Tuberculin is a very valuable diagnostic and can be of the greatest service in the fight against tuberculosis. There is no ground for warring against its general use for fear of its aggravating the disease already present." (Ninety-eight votes for it.)

In like manner a separate motion of Nocard's was carried, which was as follows:

"The Congress utters the wish that the governments may provide for the use of tuberculin in the herds where tuberculosis is found." (Seventy-three votes for it.)

The following motions were lost:

"Tuberculin is a valuable diagnostic agent. The Congress recommends the method of fighting tuberculosis which has been applied in Denmark." (Arloing, Perrencito for.)

"The Sixth International Veterinary Congress recognizes tuberculin as the best means at present of recognizing tuberculosis, and consequently as the best means of fighting the disease. It expresses the wish that the use of tuberculin by the veterinary police of the different States be regulated by law." (Butel for.)

"The Sixth International Veterinary Congress takes cognizance of the attempts of Denmark to fight cattle-tuberculosis, which have been carried on with great knowledge and accompanied by the best results by the application of tuberculin, and asks the federative council of Switzerland to communicate the action of this assembly in regard to the subject to all governments, and to recommend the general application of the Danish method to a thorough trial." (Feser for it.)

At the third session, September 18th, Raupach presided. Strebel and Hess brought in reports in regard to inoculation for symptomatic anthrax, whereupon a motion of Kitts was passed:

"The Veterinary Congress considers the preventive inoculation for symptomatic anthrax, which has been discovered by Arloing, Cornevin, and Thomas, as a very valuable prophylactic means for reducing the number of cases of symptomatic anthrax, which appears very feasible under the circumstances, that provision be made for indemnifying losses through cases of this disease."—*Thierärtz Centralblatt*, October 15, 1895.

Number of Public Slaughter-houses in the Kingdom of Prussia. On the 31st of March, 1894, there were in existence public slaughter-houses in the following districts: Königsberg, 18; Gurnbeinen, 13; Dantzic, 4; Marienwerder, 16; Berlin, 1; Potsdam, 9; Frankfurt (on the Oder), 8; Stettin, 5; Köshn, 8;

Stralsund, 4; Posen, 16; Bromberg, 14; Breslau, 16; Liegnitz, 14; Oppeln, 20; Magdeburg, 6; Meiseburg, 6; Erfurt, 2; Schleswig, 1; Hanover, 1; Hildesheim, 6; Lüneburg, 3; Osnabruck, 2; Aurich, 2; Münster, 6; Minden, 7; Arrisberg, 19; Cassel, 11; Wiesbaden, 4; Koblenz, 5; Düsseldorf, 15; Cologne, 5; Treves, 5; Archen, 3; and Sigmaringen, 2. In all 35 districts and 277 slaughter-houses.—*Thierärtz Centralblatt*, October 1, 1895.

The Question of the Introduction of Compulsory Meat-Inspection and the Insurance of Cattle by the State in Germany. The suggestion of the Chamber of Commerce in Zettan to the Bundesrath (Federative councilman), that the compulsory inspection of meat should be demanded for the whole Empire, has been the cause of a closer consideration of this question in Munich. The committeeman, Heiler (to whom the question was referred), holds that a satisfactory meat-inspection is only possible if the importation of slaughtered animals from abroad is prohibited. In the year 1894 a fifth of all the meat sold in Berlin is said to have been imported from abroad, and in other cities, such as Leipzig, the same is the case. Meat which is imported from abroad cannot be satisfactorily examined. Meat-inspection in foreign lands cannot be carefully criticised, and therefore forms no adequate equivalent for inspection at home. Imported meat which is on sale in the home market, in spite of inspection, does not offer the guarantees that cattle do which are killed in slaughter-houses under veterinary inspection. Therefore, the importation of living animals should be permitted free of duty, or at least no very great obstacles should be put in its way. The committeeman thought it best that the individual States should each for itself regulate the meat-inspection. At present, even in States where meat-inspection is practised, there is a great lack, especially in the country, of meat inspectors who are competent to perform their duties with intelligence. The recommendation of compulsory cattle-insurance by the state was also passed by the Munich Chamber of Commerce on the recommendation of Heiler.—*Thierärtz Centralblatt*, October 1, 1895.

The Apothecaries against the Veterinarians' Right to Dispense Drugs. Once more the question of the liberty of the veterinarians to dispense medicines has been brought upon the carpet by certain apothecaries, who imagine that their business is injured by it. First, an apothecary, Schlegel, in Haida, brought a motion before the annual meeting of the Bohemian Society of Apothecaries to prohibit (if possible, by legal measures) homœopathic physicians and veterinary surgeons from dispensing medicine, and later Apothecary Janota, of Falkenau, brought the same motion before the general assembly of the Austrian Pharmaceutical Society in Karlsbad. Naturally Janota's motion was supported by Schlegel, and it was carried by a large majority.—*Thierärtz Centralblatt*, October 1, 1895.

Regulation of the District Government in Regard to the Consumption of Meat and Fat which Sticks to Green Hides. In regard to the circumstance that this hide-meat, partly on account of its origin and partly on account of the qualities which it gets through transportation and storage, is at least of very doubtful character for the health of the community, the government found it necessary to make the following law:

The use of this hide-meat or of the fleshy and fatty parts which stick to green hides is prohibited, either as food for human consumption or for the feeding of domestic animals, from the time that the hides are brought from the slaughter-houses to the tanneries or become in general an article of merchandise. Offences against the law are punished by a fine of from 1 to 100 florins or with arrest and imprisonment from six hours to fourteen days.—*Thierärtz Centralblatt*, October 1, 1895.

The Applicability of Barium Chloride in the Treatment of Colic in the Horse. We take the liberty of offering some communications on the action of chlorobarium which are taken from articles by Professor Dieckerhoff in Nos. 23, 24, 27, and 29 of the *Berliner Thierärztlichen Wochenschrift*, 1895 (*Berlin Veterinary Weekly*):

"Exceedingly interesting is the manner in which Dieckerhoff learned of the action of this medicine. Two horses which were used for hauling coal from one of the depots in Berlin were left standing before a freight car which had shortly before been unloaded. In this car was a salty mass which the horses licked up. In the course of half an hour when the horses had returned from the depot they were both taken sick with colic, which took an uncommonly severe and peculiar course, accompanied by violent diarrhœa, ending fatally in the first case within an hour, in the second within fourteen hours.

"The veterinary surgeon called to treat the cases, on account of the peculiar symptoms, called in Professor Dieckerhoff, who held a post-mortem examination. He found in the stomachs a moderate quantity of food-matter. The small and large intestines, however (the mucous membrane of which was of a gray color and in places, particularly at the folds, inflamed and swollen), were completely empty and looked as if they had been washed out. The muscles of the heart appeared parenchymatously degenerated and contained a moderate quantity of tar-like, slightly coagulated blood; no other changes worthy of mention were noticeable. A chemical analysis showed a relatively large quantity of barium chloride in the contents of the stomach. The salts, also, which were afterward taken from the freight car mentioned above proved to be chlorobarium. It was then clear that this matter had brought about fatal poisoning in the two horses and had in each case exercised an uncommonly powerful action on the peristaltic action of the stomach.

"With this observation as a starting-point, Dieckerhoff made several searching experiments, the results of which are given below. This medicine given through the mouth in doses of from 2 to 6 g. had no visible effect upon a horse; after doses of 12 g. the horses showed symptoms of a light colic, and after thirty to forty-five minutes copious evacuations followed, which consisted at first of pulpy and later of fluid excrement. These evacuations were accomplished by a violent pressure, so to speak, and from the acid odor of and the undigested condition of the pulpy food-stuff there could be no doubt that the contents of the small intestine also had been eliminated. The same horses showed an unpleasant sensation of taste after the administration of barium chloride.

"The effect of chlorobarium which has just been described Dieckerhoff witnessed further in horses which were suffering from colic caused by overfeeding with corn and peas; restlessness in the animal always increased, but after twenty-five or thirty minutes a rich evacuation of excrement followed, and after several hours the horse could be left as cured of colic. A much more precise effect and in the space of two to three minutes is obtained through intravenous administration in doses from 1.0 to 1.5 g., and shows itself in that contraction of the lips, which indicates an unpleasant sensation of taste, in the raising of the tail, in colicky symptoms, and in copious evacuation of excrement. In larger doses it causes continuous cramp-like contractions of the intestines and severe diarrhœa, and soon becomes, on account of its inhibitory influence on the spinal cord, a deadly poison.

"Subcutaneous application does not bring the wished-for action in the peristalsis of the intestines, and is also dangerous for the reason that at the place of injection mortification of the skin sets in.

"Cattle can stand chlorobarium up to 40 g. through the mouth, and 3 g. intravenously without any special effect. Sheep died after an administration of 6 g., showing lameness and diarrhœtic evacuations.

"Chlorobarium dissolves in 2½ parts of cold and 1½ parts of boiling water. The same can be applied in doses of from 5 to 12 g. in solution, or with pulv. natr. chlor. 100, pulv. rad. althæ 40, and aqua destill. q. s. in electuarium or in bolus form, and finally for intravenous injection in doses of 0.5 to 1.25 g. dissolved in 10 g. aqua destill.

"However, the combination of barium chloride with sulphuric acid salts is always to be avoided, because the insoluble and useless barium sulphate is formed; natrium sulphate is therefore to be used as antidote in case of intoxication by barium chloride. Dieckerhoff prefers the intravenous injection on account of the immediate action of the drug. The dose, however, should never be repeated within twelve hours. The application of an intravenous injection, after which the animal should be exercised for a half-hour on account of the increasing restlessness, is, according to Dieckerhoff, a comparatively easy matter, and, by observing the necessary foresight, an operation without danger, which the practical veterinarian can perform just as easily as a subcutaneous injection.

"Dieckerhoff emphasizes the fact that the wise administration of barium chloride is of the highest importance if no circumstances are present which endanger the life of the animal. Of course, here the size and weight of the animal, together with his age and physical condition, are to be taken into consideration. Small and light animals, or old and emaciated ones, should be given only small doses. Especially if there is heart weakness in the animal (over seventy beats in the minute), only a very small dose—from 0.3 to 0.75 g.—dare be given.

"Chlorobarium is a deadly poison for human beings, and must be handled with great caution.

"Finally, we should mention that a syringe (capable of holding 20 g.), two canulas, and five small glasses for chlorobarium à 0.50, 0.75, and 1 g., have been prepared by the instrument manufacturer Hauptner, in Berlin, N. W., for intravenous chlorobarium injections."

